# Comprehensive assessment of antioxidant, antidiabetic, and anti-glycation properties of aqueous and methanolic extracts from *Pistacia lentiscus* L. leaves: a potential natural source for managing oxidative stress and diabetes-related complications

**DOI:** 10.3389/fphar.2025.1551841

**Published:** 2025-03-21

**Authors:** Hasnae El Allaoui, Khadija Haboubi, Kawthar El Ahmadi, Mohamed Bouhrim, Aouatif ElAbdouni, Bruno Eto, Abdelaaty A. Shahat, Rashed N. Herqash, Mohmed El Bestrioui, Zakia Zouaoui, Mohamed Nhiri

**Affiliations:** ^1^ Laboratory of Engineering Sciences and Applications, National School of Applied Sciences of Al Hoceima, Abdelmalek Essâadi University, Al-Hoceima, Morocco; ^2^ Biological Engineering Laboratory, Faculty of Sciences and Techniques, Sultan Moulay Slimane University, Beni Mellal, Morocco; ^3^ Laboratoires TBC, UFR3S, Département de Pharmacie, Université de Lille, Lille, France; ^4^ Department of Pharmacognosy, College of Pharmacy, King Saudi University, Riyadh, Saudi Arabia; ^5^ Laboratory of Biochemistry and Molecular Genetics, Faculty of Sciences and Technologies of Tangier, Tangier, Morocco

**Keywords:** *Pistacia lentiscus* L., diabete mellitus, oxidative stress, medical herbes, phytochemical screening, antidiabatic activity, antiglycation activity

## Abstract

This study evaluates the phenolic and flavonoid contents, as well as the antioxidant, antidiabetic, and anti-glycation properties of aqueous and methanolic extracts from *Pistacia lentiscus* L. leaves. The antioxidant activity was assessed using DPPH, ABTS, FRAP, and iron-chelation assays, revealing superior activity in the aqueous extract. Both extracts exhibited potent antidiabetic effects by inhibiting the digestive enzyme alpha-amylase, with IC50 values of 2,291 ± 0.002 μg/mL (aqueous) and 2,889 ± 0.002 μg/mL (methanolic). Additionally, the extracts demonstrated significant anti-glycation activity, reducing advanced glycation end-product (AGE) formation, inhibiting fructosamine levels, and protecting thiol groups, with the aqueous extract providing greater protection. These findings underscore the potential of *P. lentiscus* L. as a natural source of bioactive compounds for managing oxidative stress and diabetes-related complications.

## 1 Introduction

Diabetes mellitus is one of the most prevalent chronic non-infectious diseases globally, recognized as a leading cause of mortality and morbidity due to its associated complications. The condition arises when the pancreas fails to produce sufficient insulin or when the body cannot effectively use the insulin produced, resulting in hyperglycemia; a buildup of glucose in the bloodstream (mondiale de la Santé, 2016). Persistent hyperglycemia can lead to severe complications, including nephropathy, neuropathy, retinopathy, angiopathy, cardiovascular diseases, and certain cancers, making type 2 diabetes a significant public health concern (Morton et al., 2020).

Hyperglycemia, the primary metabolic disorder in diabetes, triggers non-enzymatic glycation, wherein monosaccharides like glucose bind to protein amino groups, forming glycated proteins such as glycated hemoglobin. This marker is widely used for assessing long-term glycemic control in diabetic patients (Gillery, 2006; Schlienger, 2013). The glycation process results in the formation of advanced glycation end products (AGEs), which impair protein structure and function. These AGEs, resistant to degradation, contribute to the onset and progression of diabetic complications (Dali-Youcef, 2010). Moreover, glycation is intricately linked with oxidative stress, collectively termed “glycoxidation,” where both processes generate reactive oxygen species (ROS), exacerbating cellular damage (Florence, 1995) Different systems, such as enzymes or antioxidant molecules like tocopherol, ascorbate, carotenoids, and polyphenols, can scavenge free radicals and restore oxidative balance. However, an excess of free radicals not neutralized by the body’s defense mechanisms can lead to oxidative stress (Barouki, 2006). There are several pharmaceutical drugs available, such as Voglibose and acarbose, that are used to control postprandial hyperglycemia in diabetic patients. However, the long-term use of these drugs may carry certain undesirable side effects include hepatotoxicity, gastrointestinal symptoms, and cardiovascular problems (Chaudhury et al., 2017).

Herbal medicine has reemerged as a potential strategy to prevent or delay complications associated with oxidative stress and hyperglycemia. Medicinal plants provide a rich source of bioactive compounds with multi-target effects, offering low toxicity and minimal side effects, making them promising candidates for natural diabetes management. Among these medicinal plants, *Pistacia lentiscus* L. *(P. lentiscus)*, a member of the Anacardiaceae family, is widely distributed in the Mediterranean region, spanning North Africa, Southern Europe, and parts of Asia, and extending to the Canary Islands. Commonly known as mastic tree, it is a slow-growing, evergreen shrub, reaching 1–8 m in height, depending on environmental conditions. It is characterized by its dark green, leathery leaves, red to black drupes, and apetalous flowers, with flowering occurring from March to May. The species is distinguished from other Pistacia trees by its paripinnate leaves, which end in a pair of leaflets rather than a single one (Mansour-Djaalab et al., 2012; Bozorgi et al., 2013; Bammou et al., 2015).

This species has a long history of medicinal, industrial, and nutritional applications. All parts of the plant, including the leaves, bark, resin, and seeds—have been utilized in traditional medicine across the Mediterranean basin. In North African folk medicine, its leaves are commonly prepared as infusions and decoctions to treat digestive disorders, respiratory ailments (asthma, bronchitis), skin infections (eczema, oral ulcers) and headaches (Benhammou et al., 2008; Golan-Goldhirsh, 2009). Despite its wide traditional use, modern pharmacological studies on *P. lentiscus* have primarily focused on its essential oil and resin, which exhibit antibacterial, anti-inflammatory, and gastroprotective properties (Derwich et al., 2010). However, the pharmacological potential of its leaf extracts remains significantly underexplored, particularly in the context of diabetes, oxidative stress, and protein glycation inhibition. Phytochemical studies have revealed that *P. lentiscus* leaves are rich in flavonoids (quercetin, myricetin), phenolic acids (gallic acid, syringic acid), tannins, and terpenoids, all of which have been associated with antioxidant and enzyme-inhibiting properties (Floris et al., 2024). Numerous studies have explored plant-based inhibitors of α-amylase and glycation, particularly within the *Pistacia* genus. For instance, previous research on *Pistacia vera* L.demonstrated its ability to inhibit advanced glycation end products (AGEs) and oxidative stress through its rich polyphenolic composition. particularly flavonoids and tannins. Similarly, studies on *Pistacia atlantica* Desf. have reported significant antioxidant and anti-diabetic properties, attributed to its high phenolic content and enzyme inhibitory activity (Atta et al., 2023). In contrast, research on *P. lentiscus* remains limited, particularly regarding its ability to modulate carbohydrate metabolism, inhibit α-amylase, and prevent protein glycation. While some studies have investigated its antioxidant and antibacterial activities, there is scarce scientific evidence supporting its efficacy as an anti-diabetic or anti-glycation agent. Given the structural diversity of its polyphenolic and flavonoid compounds, *P. lentiscus* could serve as a natural inhibitor of oxidative stress and glycation, both of which play key roles in diabetes complications (Floris et al., 2024). The pharmacological properties of *P. lentiscus* have been widely recognized in traditional medicine, yet its role in diabetes management remains underexplored. This study aims to provide a comprehensive assessment of the phytochemical composition and biological properties of *P. lentiscus* leaves, focusing on their antioxidant, antidiabetic, and anti-glycation potential. Specifically, we evaluate the total phenolic and flavonoid content using spectrophotometric methods, followed by antioxidant capacity assays (DPPH, ABTS, FRAP, and iron-chelating ability) to determine the extract’s free radical scavenging and reducing power. To investigate its potential in diabetes management, α-amylase inhibition assays are performed to assess its ability to regulate carbohydrate metabolism and control postprandial glucose spikes. Additionally, the anti-glycation effects of *P. lentiscus* extracts are examined through multiple *in vitro* assays, including AGE inhibition, fructosamine reduction, and thiol group protection. The phytochemical composition of the extracts is further analyzed using HPLC-DAD to identify key bioactive compounds responsible for these biological effects. While previous studies have highlighted the antioxidant and antibacterial activities of *P. lentiscus* (Floris et al., 2024), its anti-glycation and enzymatic inhibition potential remain largely uninvestigated. To our knowledge, this is the first study to systematically evaluate the multi-target mechanisms of *P. lentiscus* in oxidative stress and diabetes management. By bridging this knowledge gap, the present findings not only provide scientific validation for its traditional use but also position *P. lentiscus* as a promising natural candidate for future nutraceutical and pharmaceutical applications.

## 2 Materials and methods

### 2.1 Plant material

*P*. *lentiscus* leaves were collected from the region of Tazorakht Al Hoceima in Morocco in March 2023. The geographical coordinates of the collection site were recorded using a global positioning system (GPS) as 35°00′39.4″N 3°51′19.2″W. After collection. The plant leaves were washed, dried at room temperature (RT) for 15 days in a dark place, ground into powder, and stored in a tightly sealed container protected from light to preserve their integrity and moisture until further use.

### 2.2 Preparation of plant extracts

The plant powder was extracted by maceration with either distilled water or methanol 80% (45 mL/4.5 g of plant powder) at RT and in dark conditions with continuous shaking for 48 h until the extracts appeared slightly turbid in the siphon tube. Afterward, the mixture was centrifuged at 8,000 rpm for 10 min, and the resulting supernatant was filtered through a Whatman filter paper. The water and methanol solvents were evaporated to dryness in an incubator at 40°C for 48 h. The obtained extracts were stored at 4°C until further use. The extraction yield was calculated using the following formula:

$$\text{yield}\left(\%\right)=\frac{w_{2}-w1}{w0}\times 100$$

where.

W2: the weight of the extract and the container,

W1: the weight of the container alone,

W0: the weight of the initial dried sample.

### 2.3 Phytochemical analysis

#### 2.3.1 Determination of total phenolic content

The total phenol contents were determined by spectrophotometry referred to the method of Folin-Ciocalteu (FC) as described by M. El bastrioui (El bastrioui et al., 2022). In brief, for each repetition, 100 µL of each extract was mixed with 0.4 mL of the FC reagent. Then, to the mixture, 0.1 mL of deionized water (dH2O) and 1 mL of sodium carbonate solution [Na_2_CO_3_ prepared at a concentration of 7% (w/v)] were added. After incubation in the dark at room temperature for 30 min, the absorbance was measured at 725 nm using a UV spectrophotometer. The results were expressed as milligrams of Gallic acid equivalent per Gram of dry extract weight.

#### 2.3.2 Determination of total flavonoid content

The total flavonoid content in *P. lentiscus* leaf extracts was determined using a colorimetric method by adding aluminum chloride reagent (AlCl3). Following the protocol described by Ben Mrid et al. (2019), with slight modifications, 40 µL of each extract to be analyzed were mixed with 10 µL of 1 M potassium acetate, 100 µL of 50% methanol, and 10 µL of 10% (w/v) AlCl3 and the total volume was made up to 400 µL with distilled water. The resulting mixtures were agitated and incubated in the dark at room temperature for 30 min. The absorbance was measured at 415 nm, and the concentrations of flavonoids were calculated from a standard curve and expressed as milligrams of quercetin equivalent per Gram of extract.

#### 2.3.3 HPLC/UV analysis of *P. lentiscus* extracts

The HPLC analysis of *P. lentiscus* extracts was performed using a high-performance liquid chromatography system equipped with a C18 reverse-phase column (150 mm × 4.6 mm, 5 µm particle size) maintained at ambient temperature. A binary solvent system was employed for the separation, consisting of water with 0.1% formic acid (Solvent A) and acetonitrile with 0.1% formic acid (Solvent B). Solvent A was prepared by mixing 0.3 mL of formic acid with 299.7 mL of deionized water, and Solvent B was similarly prepared using acetonitrile. The chromatographic conditions included a flow rate of 1.0 mL/min with a gradient elution program: 5% Solvent B at 0 min, 10% at 5 min, 50% at 15 min, and 100% at 30 min. The sample solution (5 mg/mL) was prepared by dissolving 25 mg of extract in 5 mL of 20% methanol, and the injection volume ranged between 10 and 20 µL. Detection was performed using a diode array detector set at 250 nm to ensure precise identification of the bioactive components (Qabaha et al., 2016).

### 2.4 Antioxidant activities

#### 2.4.1 DPPH radical scavenging activity assay

The DPPH radical scavenging activity was measured using the method described by M. El bastrioui. (El bastrioui et al., 2022), with some modifications. In tubes, 15 μL of each extract at different concentrations were introduced with 185 μL of a DPPH solution (0.1 mM dissolved in methanol). The mixture is vigorously vortexed and left for incubation in the dark at RT for 30 min. After that, the decrease in absorbance was measured at 517 nm using a spectrophotometer. The results were expressed as a percentage of inhibition, calculated using the following equation:

$$\%\text{ scavenging effect}=\frac{\text{Abc}-\text{Abs}}{\text{Abc}}\times 100$$

Where Abc is the absorbance of the control (containing all reagents except the test compound), Abs represents the absorbance obtained from the mixture of the extract and DPPH solution after incubation. The extract concentration that provides 50% inhibition (IC50) was calculated from the graph of the scavenging effect percentage against extract concentrations in the solution.

#### 2.4.2 ABTS radical scavenging assay

The ABTS^+^ discoloration test was conducted using the colorimetric method, as described by Ben Mrid et al. (2019). Initially, 925 µL of the cationic radical ABTS^+^ was mixed with 75 µL of each extract, and the mixture was incubated at 30°C for 15 min. After incubation, the absorbance of the ABTS^+^ radical was measured at 734 nm. The percentage of inhibition was calculated using the following equation:

$$\%\text{ scavenging effect}=\frac{\text{Abc}-\text{Abs}}{\text{Abc}}\times 100$$

Where Abc is the absorbance of the control (containing all reagents except the test compound), Abs represents the absorbance obtained from the mixture of the extract and DPPH solution after incubation. The extract concentration that provides 50% inhibition (IC50) was calculated from the graph of the scavenging effect percentage against extract concentrations in the solution.

#### 2.4.3 Ferric reducing antioxidant power assay

The reduction power was determined using various concentrations of methanolic and aqueous extracts of *P. lentiscus* leaf. Following the protocol described by M. El Bastrioui (El Bastrioui et al., 2024). with some modifications. 200 μL of each extract was mixed with 500 μL of phosphate buffer (0.2 M, pH 6.6) and 500 μL of potassium ferricyanide (1%). The mixture was then incubated at 50°C for 20 min. After the incubation period, 500 μL of trichloroacetic acid (10%) was added to stop the reaction. The mixture was centrifuged at 3,000 rpm for 10 min, and then 500 μL of the supernatant was mixed with 500 μL of distilled water and 100 μL of ferric chloride (0.1%). The absorbance of the resulting solution was measured at 700 nm. The same test is conducted with a reference molecule, ascorbic acid, to create a calibration curve for calculating the equivalence in milligrams of ascorbic acid per Gram of extract.

#### 2.4.4 Iron chelating power

The iron chelating power of the leaf extracts was demonstrated using the protocol described by Dinis et al. (1994). with some modifications. In glass tubes, 800 µL of increasing concentrations of each sample were added to 50 µL of FeCl2 (2 mM). After thorough mixing, the tubes were left to stand at RT for 30 min 50 μL of ferrozine (5 mM) and 500 µL of deionized water were added to initiate the reaction. The absorbance was measured at 562 nm after incubating the mixtures for 10 min at RT, and the IC50 value was calculated from the graph of the scavenging effect percentage against the extract concentration in the solution.

### 2.5 *In vitro* antidiabetic effects

#### 2.5.1 α-amylase inhibitory assay

The α-amylase inhibitory activity was evaluated following the method described by Bouchmaa et al. (2022). In this assay, various concentrations of the sample extracts were mixed with α-amylase enzyme (0.1 U/mL) solubilized in phosphate buffer (20 mM, pH 6.9) and incubated at 37°C for 30 min. After the initial incubation, 0.25% starch solution in phosphate buffer (pH 6.9) was added to initiate the enzymatic reaction, and the mixtures were further incubated at 37°C for 30 min. After that, the reaction was stopped by adding 200 µL of DNS reagent, composed of sodium-potassium tartrate (12% in 0.4 M NaOH) and 3,5-dinitrosalicylic acid (1%). The tubes were then heated in boiling water for 5 min and cooled to room temperature. The mixture was diluted by adding 600 µL of distilled water, and the absorbance was measured at 540 nm. The inhibitory activity of α-amylase was expressed as a percentage of inhibition, calculated using the appropriate equation:

$$\text{Inhibition }\left(\%\right)=\frac{\left(\text{Ac}-\text{Acb}\right)-\left(\text{As}-\text{Asb}\right)}{\left(\text{Ac}-\text{Acb}\right)}\times 100$$

where Ac is the absorbance of the control (composed of the Enzyme only), Acb refers to the absorbance of the control blank (contains the buffer without the Enzyme), As and Asb refer to the absorbance of the sample (contains Enzyme and inhibitor), and the sample blank, (contains the inhibitor without the Enzyme), respectively.

#### 2.5.2 Antglycation activities

This assay was based on an *in vitro* model for comparing and evaluating the anti-glycation activities of methanolic and aqueous extracts of *P. lentiscus* leaves, following the method described by Mrid et al. (2022). Commercially available bovine serum albumin (BSA) was used as the protein source rather than an *ex vivo* isolated form. Fructose was dissolved in a phosphate buffer solution (0.1M, pH 7.4) to prepare a 500 mM solution. Afterward, BSA (10 mg/mL) was added to the fructose solution and incubated at 37°C for 4 weeks in the presence and absence of 60 µL of various concentrations of the sample extracts. To prevent microbial growth, 0.05% sodium azide was used as an inhibitor. The quantification of advanced glycation end products (AGEs) was performed using a spectrofluorometer with an excitation wavelength of 355 nm and an emission wavelength of 460 nm. A positive control (Aminoguanidine, AG) was included in the study to compare inhibition levels. The inhibition rates of AGE formation were determined using the following equation:

$$\%\text{ inhibition}=\left[1-\frac{\left(\text{FLs}-\text{FLsb}\right)}{\left(\text{fLc}-\text{FLcb}\right)}\right]\times 100$$

FLs represents the mixture’s fluorescence intensity, FLsb corresponds to the fluorescence intensity of the sample blank (without Fructose), FLc is the fluorescence intensity of the control mixture, and FLcb refers to the fluorescence intensity of the control blank combination.

#### 2.5.3 Determination of fructosamine

The fructosamine assay was performed after 4 weeks of incubation, following the protocol described by Zhang et al. (2021). Briefly, 200 µL of glycated substance was mixed with 800 µL of Nitro Blue Tetrazolium NBT (0.3 mM) solubilized in a sodium carbonate buffer (100 mM, pH 10.35). After that, the resulting mixture was incubated at 37°C for 30 min. The Fructosamine reduces NBT and produces coloration, which has an absorption at 530 nm. The percentage of inhibition fructosamine was determined using the following equation:

$$\%\text{ inhibition}=\left[1-\frac{\left(Aglycated\right)}{\left(\text{Ablank}\right)}\right]\times 100$$

Where A glycated is the absorbance of the glycated substance and A blank is the absorbance of the blank solution.

#### 2.5.4 Determination of protein thiol group

The fructosamine content in glycated materials was determined using Ellman’s reagent after 4 weeks of incubation (Bouchmaa et al., 2022). Briefly, 250 µL of glycated samples were incubated for 15 min at 37°C after adding 750 µL of DTNB solution (0.5 mM) prepared in sodium phosphate buffer (100 mM, pH 8), and the absorbance was measured at 410 nm. The following formula is used to estimate the percentage protection of thiol groups in the sample:

$$\%\text{ Protection}=\left[\frac{\left(\text{Asample}-\text{Ablank}\right)}{\left(\text{Acontrol}-\text{Ablank}\right)}\right]\times 100$$

Where Asample refers to the absorbance obtained from the glycated samples incubated with DTNB solution, while Ablank represents the absorbance of the blank sample containing all reagents except the test sample, the Acontrol refers to the absorbance obtained from a control sample without glycated samples.

### 2.6 Statistical analysis

*In vitro* results were obtained from three independent experiments and were expressed as mean ± standard deviation (SD). One-way analysis of variance (ANOVA) was used to determine the statistically significant results (*p* < 0.05), using version 18 of SPSS statistics.

## 3 Results

### 3.1 Total phenolic, flavonoid in the methanolic and aqueous extract

Polyphenols are a highly abundant and widely distributed class of bioactive compounds in the kingdom of Plantae. They can be classified into two major groups: flavonoids and non-flavonoids (phenolic acids). Plants produce them as part of their secondary metabolites in response to various stimuli such as infections, injuries, exposure to ultraviolet radiation, and insect infestation. These compounds have gained significant attention due to their natural antioxidant properties and are increasingly known for their potential to prevent and treat various diseases (Chira et al., 2008; El Allaoui et al., 2024a).The phytochemical screening results of methanolic and aqueous extract in the *P. lentiscus* leaves are shown in Table 1.

**TABLE 1:** The amount of polyphenols and flavonoids content in P. lentiscus leaves depending on the extraction solvent and the yield.

	Flavonoids mg EQ/g DW	Polyphenol mg EAG/g DW	Yield extract %
Methanolic extract	82,550 ± 3,841^a^	344,821 ± 2,301^a^	24,66^a^
Aqueous extract	98,597 ± 2,779^b^	385,730 ± 6,624^b^	20,06^b^

The values of total phenolic compounds were expressed in milligrams of Gallic acid equivalents (EAG) per Gram of dry weight (DW) of the sample. The results were expressed in milligrams of quercetin equivalents (EQ) per Gram of dry plant for flavonoids. Different letters indicate significant differences between conditions (p < 0.05).

**FIGURE 1:**
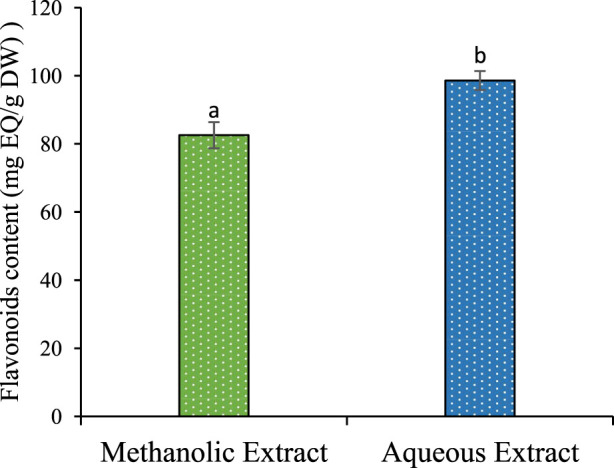
Polyphenol content of methanolic and aqueous extract for P. lentiscus. Each value represents the average of three replicates. The bars represent the type of error. Different letters indicate significant differences between conditions (p < 0.05).

**FIGURE 2:**
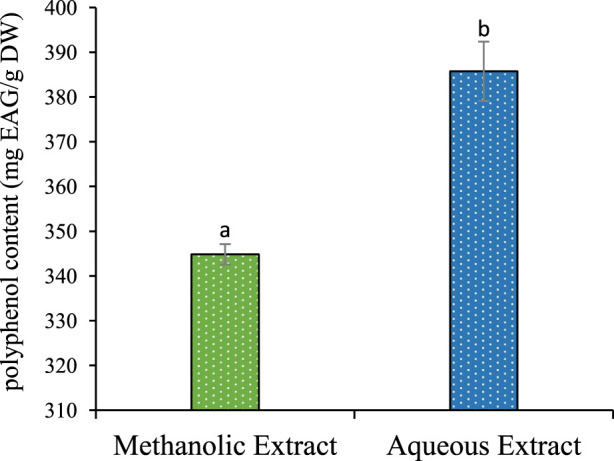
Flavonoids content of methanolic and aqueous extract for P. lentiscus. Each value represents the average of three replicates. The bars represent the type of error. Different letters indicate significant differences between conditions (p < 0.05).

The results described in Table 1 reveal a significant influence of the extraction power of the solvent on the yield. Methanol gives a better yield of 24.66%, and water yields 20.06%. The phenolic and flavonoid contents in the aqueous extract of *P. lentiscus* leaves were found to be (385,730 ± 6,624 mg GAE/g DW) and (98,597 ± 2,779 mg EQ/g DW) (Figure 1). respectively, which are significantly (p < 0.05) higher than the phenolic and flavonoid content of the methanolic extract (344,821 ± 2,301 mg GAE/g DW) and (82,550 ± 3,841 mg EQ/g DW) (Figure 2), respectively. If we compare the values of the flavonoid contents with those of the phenolic compound contents (Table 1), we observe that the flavonoid contents are lower than the phenolic compound; this suggests that the extracts contain author phenolic compounds with different chemical structures, such as phenolic acids, tannins, and stilbenes.

Comparing our results with the literature, the phenolic compound content in *P. lentiscus* leaves in our study is lower than that reported in the recent research by Dahmoune et al. (2014) (632.9 ± 1.35 mg GAE/g DW). However, another study conducted by Barbouchi et al. (2020) showed a slight similarity with an average content of (345.95 ± 1.17 mg GAE/g DW). On the other hand, our extract has a higher content of flavonoids compared to the content found in the recent studies conducted by Dahmoune et al. (2014) (38.7 mg EQ/g DW).

### 3.2 Qualitative identification of chemical composition of extracts

**FIGURE 3:**
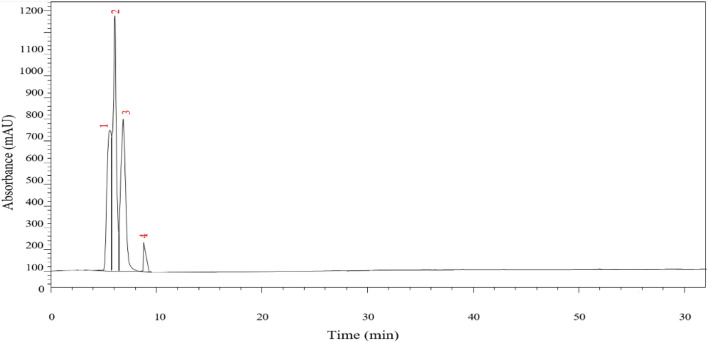
Chromatograms of the P. lentiscus leaves at 250 nm.

The chemical profile of *P. lentiscus* extract was investigated using HPLC-DAD at a detection wavelength of 250 nm, highlighting the presence of several phenolic compounds known for their potent antioxidant properties. The chromatographic analysis identified four prominent peaks attributed to gallic acid, quercetin, catechin, and myricetin (Figure 3; Table 2). These identifications were achieved by aligning the retention times of the detected peaks with those of authentic reference standards. Additionally, the retention times and corresponding compound assignments were corroborated through a detailed comparison with prior studies on *P. lentiscus* and standardized HPLC profiles of phenolic compounds. This robust analytical approach reinforces the reliability and accuracy of the findings, providing valuable insights into the bioactive constituents of the extract.

**TABLE 2:** Phenolic compounds of the aqueous extracts from P. lentiscus Leaves.

Peak N°	Presumed compound	Retention time (Rt, min)	Approx % area	References study
1	Gallic acid	5.2	15.20%	Qabaha et al. (2016)
2	Quercetin	6.8	45.80%	Bouakline et al. (2024)
3	Catechin	7.5	25.40%	Bouakline et al. (2024)
4	Syringic acid	8.9	13.60%	Mehenni et al. (2016)

Quercetin emerged as the dominant compound, contributing to its well-documented therapeutic effects, including antioxidant and antidiabetic activities. This was followed by catechin and gallic acid, which further support the bioactive potential of *P. lentiscus*. These results align with previous research emphasizing the plant’s richness in bioactive compounds. For instance, Qabaha et al. (2016). Identified gallic acid as a significant phenolic acid in *P. lentiscus* leaves, while quercetin and catechin were reported in another study by Bouakline et al. (2024) Additionally, syringic acid was identified in a study by Mehenni et al. (2016) further corroborating our findings.

### 3.3 Antioxidant activity

Oxidation is a vital process that occurs during the metabolism of aerobic cells in the body, and it involves the oxygen molecule. Uncontrolled oxygen production through metabolic processes can form free radicals, which are known to contribute to oxidative stress (Southorn and Powis, 1988). The generation of radicals can be accelerated by several factors, including transition metals such as Iron and copper; these metals are rarely found in the free state of the body. Often carrying different organic molecules, for example, transferrin, ferritin, ceruloplasmin, etc (Krinsky, 1992). Free radicals and transition metals can be eliminated or delayed by natural molecules such as polyphenols and flavonoids, and these compounds can inhibit the rate of oxidation and protect cells from damage (Charrier and Anastasio, 2011). As a result, they may contribute to reducing the risk of certain chronic diseases such as diabetes and cancers. plants with high phenolic components can be a great source of antioxidants (Khokhar et al., 2003). This study aims to highlight the antioxidant activity of *P. lentiscus* leaves by four methods: scavenging free radical DPPH and ABTS, reducing power, and iron chelating power. The results of the four tests are shown in Table 3:

**TABLE 3:** Values (mean ± SD) obtained in the antioxidant activity assays of the P. lentiscus leaf extracts.

Antioxidant capacity (IC_50_ values; mg/mL)				
	DPPH scavenging	ABTS scavenging	Iron chelation power	Reducing Power (mg AAE/g DW
Methanolic extract	0.047 ± 0,001^a^	0.498 ± 0,050^a^	1,27 ± 0,004^a^	596,130 ± 5,555^a^
Aqueous extract	0.037 ± 0,003^b^	0.124 ± 0,004^b^	0,47 ± 0,001^b^	597,826 ± 6,282^a^
	BHA: 0.0637 ± 0.0116^c^	BHA: 0.0013 ± 0.00008^c^	EDTA: 0.296 ± 0.0006^c^	

The values are the mean of three determinations ±standard error. IC50: the concentration of the extract providing 50% inhibition; DPPH: 2,2-diphenyl-1-picrylhydrazyl; ABTS: 2,2′-casino-bis (3-ethylbenzothiazoline-6-sulfonic acid); AAE: ascorbic acid equivalent; DW: dry weight; BHA: butylated hydroxyanisole; EDTA: Ethylene Diamine Tetra Acetic. Values in the same row not sharing a common letter (a to b) are significantly different at p < 0.05.

**FIGURE 4:**
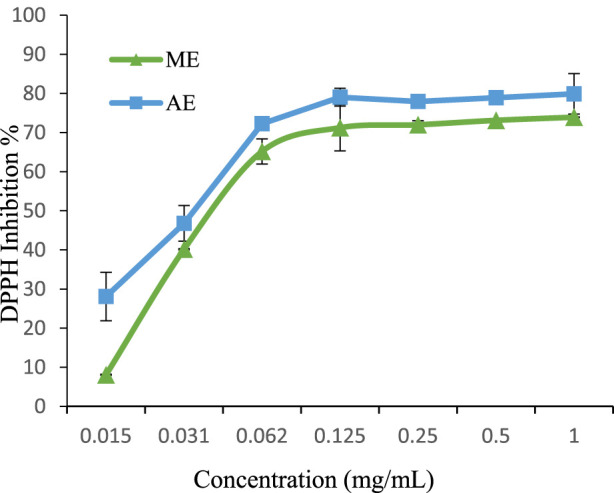
DPPH radical scavenging activity of extracts. The values are the mean of three determinations ±standard error. ME: Methanolic extract, AE: Aqueous extract.

In the present study, both methanolic and aqueous extracts of *P. lentiscus* exhibited strong DPPH scavenging activity, even at low concentrations (Figure 4). The aqueous extract demonstrated superior antioxidant capacity (IC_50_ = 0.037 mg/mL) compared to the methanolic extract (IC_50_ = 0.047 mg/mL). Notably, both extracts outperformed the synthetic antioxidant BHA (IC_50_ = 0.0637 mg/mL) (Li et al., 2013), with the aqueous extract exhibiting the most potent effect. Previous studies have also highlighted the higher DPPH scavenging potential of aqueous extracts, with reported IC_50_ values ranging from 0.09 mg/mL for aqueous extracts to 1.13 mg/mL for methanolic extracts (Barbouchi et al., 2020).

**FIGURE 5:**
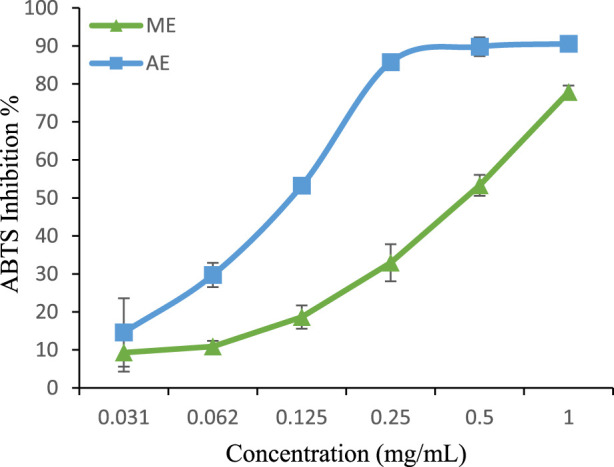
ABTS radical scavenging activity of extracts. The values are the mean of three determinations ±standard error. ME: Methanolic extract, AE: Aqueous extract.

For ABTS + scavenging activity, the aqueous extract (IC_50_ = 0.124 mg/mL) again exhibited greater radical-quenching ability than the methanolic extract (IC_50_ = 0.498 mg/mL) (Figure 5). However, both extracts were significantly less effective than BHA (IC_50_ = 0.0013 mg/mL) (Li et al., 2013), reinforcing the superior efficiency of synthetic antioxidants in this assay.Interestingly, our findings contrast with those reported by Belhachat et al. (IC_50_ = 9.48 mg/mL for methanolic and 12.64 mg/mL for aqueous extracts) (BELHACHAT, 2019), where the methanolic extract exhibited greater ABTS + scavenging activity. These variations could be attributed to differences in geographical origin, environmental conditions, and extraction methods, which influence polyphenol composition.

The reducing power of a compound is a key indicator of its antioxidant potential. As shown in Table 3, both aqueous and methanolic extracts of *P. lentiscus* exhibited strong reducing activity, with values of 597.826 ± 6.282 mg AAE/g DW and 596.130 ± 5.555 mg AAE/g DW, respectively. The minimal difference between the extracts suggests comparable electron-donating capacity. These results are significantly higher than those reported by Salhi et al. (2019), where the Fe3+/Ferricyanide complex assay yielded 301.86 ± 0.18 mg AAE/g DW for the aqueous extract and 309.60 ± 0.12 mg AAE/g DW for the methanolic extract, further confirming the strong reducing capacity of P. lentiscus.

**FIGURE 6:**
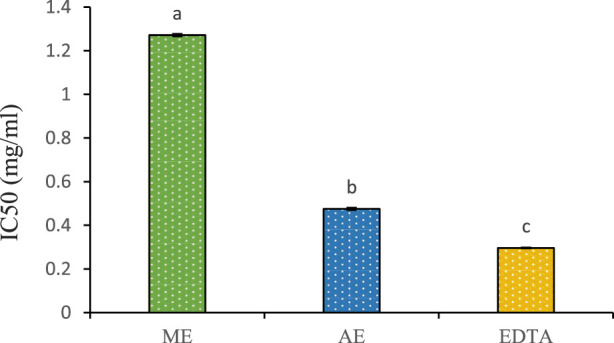
Chelating power of the aqueous (AE) and methanolic extracts (ME) of P. lentiscus. IC50: the concentration of the extract providing 50% chelation. EDTA: Ethylene Diamine Tetra Acetic. Each value represents the mean of three replicates. Bars represent the standard error. Different letters indicate significant differences among treatments at p < 0.05.

The chelating power of *P. lentiscus* leaf extracts and EDTA is presented in Figure 6. EDTA, a well-known reference compound, exhibits potent chelating activity by binding to ferrous ions and preventing their interaction with biological molecules. In this study, the aqueous extract displayed stronger chelating ability (IC_50_ = 0.47 ± 0.001 mg/mL) compared to the methanolic extract (IC_50_ = 1.271 ± 0.004 mg/mL). However, both extracts were less effective than EDTA (IC_50_ = 0.296 ± 0.0006 mg/mL), which remains the gold standard in iron chelation. Notably, the aqueous extract’s IC_50_ closely approached that of EDTA, indicating a higher affinity for ferrous ions than the methanolic extract. The chelating properties of *P. lentiscus* are likely attributed to its polyphenolic compounds, which are known to bind metal ions and facilitate their excretion from the body (Borowska et al., 2018).

### 3.4 Antidiabetic effect

#### 3.4.1 *In vitro* α -amylase inhibition assay

In diabetes mellitus, hyperglycemia leads to hyperglycation. Pancreatic and salivary α-amylases play a crucial role in catalyzing the hydrolysis of alpha-1,4-glycosidic bonds of large polysaccharides such as starch and glycogen, resulting in the formation of monosaccharides and disaccharide within the digestive system. Afterward, α-glucosidases split these monosaccharides into individual glucose molecules, easily absorbed into the bloodstream (Gillery, 2006). One of the therapeutic approaches to control hyperglycemia is to inhibit the digestion of carbohydrates. Acarbose is the most commonly used antidiabetic oral drug to delay the digestion and absorption of sugars by inhibiting digestive enzymes (Hanefeld, 1998). Therefore, inhibiting α-amylase and α-glucosidase enzymes can suppress carbohydrate digestion, slow glucose absorption, and consequently reduce blood glucose levels. In recent clinical studies, it has been shown that some natural products, such as polyphenolic compounds, exhibit strong alpha-amylase and alpha-glucosidase inhibitory effects over those of acarbose and also reduce the glycemic response to carbohydrate foods in the human body (El Allaoui et al., 2024b; Bouhout et al., 2024; Maroua et al., 2018). The present study aims to explore the α-amylase inhibition effect of *P. lentiscus* leaf extract. IC50 values of the aqueous and methanolic extract and acarbose are shown in the following Table:

To measure the inhibitory effect of each extract, we used the IC50, which represents the concentration of an inhibitor required for 50% inhibition of its targeted Enzyme. The results presented in Table 4 demonstrate the effective inhibition of α-amylase activity by both aqueous and methanolic extracts of *P. lentiscus* leaves, as indicated by their potent IC50 values. The aqueous extract has an IC50 value of 2.291 ± 0.002 μg/mL, and the methanolic extract has an IC50 value of 2.889 ± 0.002 μg/mL. However, there is no statistically significant difference between these IC50 values at p < 0.05. In contrast, acarbose exhibits the highest IC50 value of 0.9 ± 0.013 μg/mL, significantly different from the other values at p < 0.05.

**TABLE 4:** Inhibition results of P. lentiscus on α-amylase enzyme.

		IC50 (μg/mL)	
	Aqueous extract	Methanolic extract	Acarbose
*P. lentiscus*	2,291 ± 0,002^a^	2,889 ± 0,002^a^	0, 9 ± 0,013^b^

The IC50 Values are means ± standard deviation. IC50: the concentration of the extract providing 50% inhibition; Different letters (a, b) in each column indicate statistically significant differences at p < 0.05.

**FIGURE 7:**
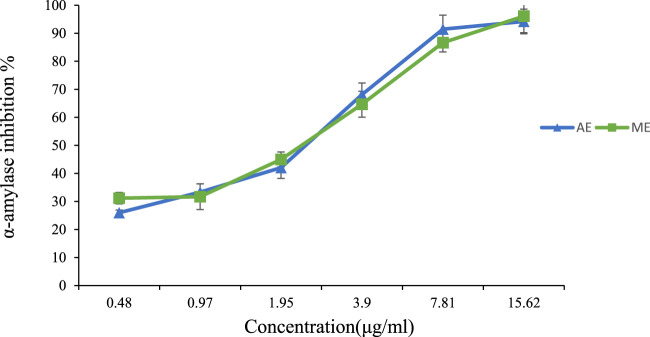
Percentage of α-amylase inhibition versus different concentrations of P. lentiscus leaves’ extract. AE: Aqueous Extract, ME: Methanolic Extract. Each value represents the mean of three replicates. Bars represent the standard error.

Figure 7 below illustrates the α-amylase inhibitory activity of both extracts across different concentrations, highlighting a dose-dependent effect. At 15.62 μg/mL, both extracts exhibited strong enzymatic inhibition, with 94% inhibition for the aqueous extract and 96% for the methanolic extract, demonstrating significant activity even at low doses. Previous studies have also investigated the α-amylase inhibitory effects of *P. lentiscus* leaf extracts. Foddai et al. (2015) reported an IC_50_ of 65.3 ± 7.4 μg/mL for the aqueous extract, while Chafiaâ et al. (Mehenni et al., 2016) found a similar IC_50_ value of 55.86 ± 7.75 μg/mL.

#### 3.4.2 Antglycation activity (anti-AGE, fructosamine inhibition, thiol group protection)

Protein glycation involves a series of complex reactions between monosaccharides and amino acids of proteins, leading to unstable Schiff bases forming an Amadori Product (AP) such as Fructosamine. During the propagation reaction, subsequent rearrangements and chemical modifications result in the formation of irreversible cross-links and the generation of stable compounds known as advanced glycation end products (AGEs) (Wu et al., 2011; Bonnefont-Rousselot et al., 2004). These reactions, known as the Maillard reaction, occur spontaneously under conditions of prolonged exposure to high glucose levels.

The accumulation of AGEs with age, particularly in individuals with diabetes, is the underlying principle for developing various complications in organs such as the kidneys, eyes, and heart. Additionally, AGEs are implicated in the pathogenesis of several diseases, including arteriosclerosis, renal failure, diabetic retinopathy, and cataracts, rendering them highly detrimental to overall health (Kandarakis et al., 2014).

Therefore, the inhibition or reduction of AGE formation and their accumulation is an essential therapeutic target in managing the complications of diabetes. Various strategies, including the utilization of AGE inhibitors and antioxidants, in conjunction with the implementation of lifestyle modifications aimed at fostering health, are being explored to reduce the harmful effects of AGEs and improve patient outcomes. Different pharmacological compounds, such as aminoguanidine, have been developed to break AGE cross-links; however, these drugs present harmful side effects (Thornalley, 2003). Recent discoveries have emerged from clinical studies and research conducted in China, Japan, and the United States, highlighting the potential of polyphenols, especially flavonoids, as potent glycation inhibitors. These compounds have shown the ability to limit the progression of detrimental mechanisms associated with the glycation process, as reported in several studies (Wu et al., 2011).

**FIGURE 8:**
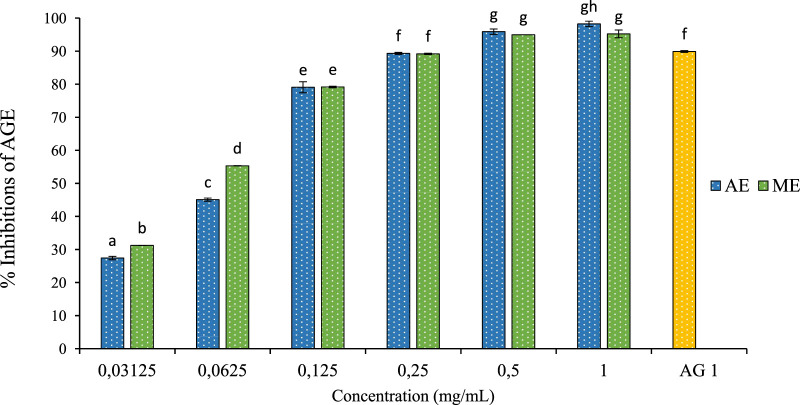
The effect of aqueous extracts (AE) and methanolic extracts (ME) of P. lentiscus on the percentage of AGE inhibition, AG: Amino Guanidine (1 mg/mL). Each value represents the mean of three replicates, and the bars represent the standard error. Different letters in each column indicate statistically significant differences at p < 0.05.

There are numerous steps in the generation of AGEs. Therefore, Antglycation functions can occur at various stages. Our study evaluated the antiglycation capacity of *P. lentiscus* leaf extracts using three tests: inhibition of AGE formation, determination of fructosamine inhibition percentage, and preservation of thiol group (R-SH). The results of these three tests are presented below:

After incubating BSA with fructose and the extract for 4 weeks, a significant reduction in the formation of AGEs was observed. As depicted in Figure 8, the extracts derived from *P. lentiscus* leaves exhibited remarkable efficacy in inhibiting AGE formation. The aqueous extract can inhibit 98.23% of AGEs, with a concentration of 1 mg/mL resulting in significant inhibition. Similarly, the methanolic extract shows inhibitory effects on 95.23% of AGEs, and 1 mg/mL of aminoguanidine also inhibits 98.88% of AGEs. These data demonstrate that these extracts have a potent dose-dependent anti-AGE activity comparable to the reference drug.

The fructosamine assay is an essential tool for evaluating early-stage protein glycation and provides insight into the short-term glycemic status of an individual. Unlike AGEs, which accumulate over time, fructosamine forms during the early stages of glycation as an Amadori product, representing a transitional phase before irreversible AGE formation (Ling et al., 2022). Measuring fructosamine levels helps evaluate the effectiveness of anti-glycation agents in preventing further glycation-related damage. Clinically, fructosamine reflects average blood glucose levels over 2–3 weeks, making it particularly useful for monitoring rapid changes in glycemic status, unlike hemoglobin A1c (HbA1c), which provides a longer-term (2–3 months) overview (Gounden et al., 2025).

**FIGURE 9:**
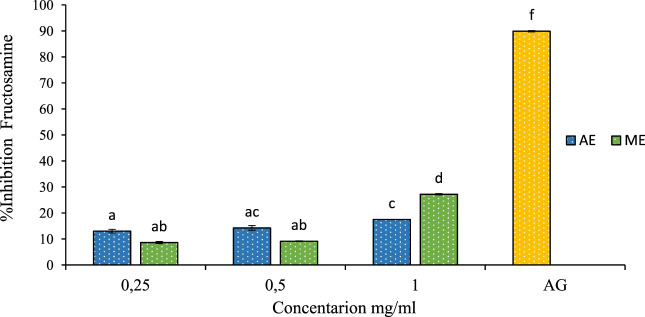
The effect of aqueous extracts (AE) and methanolic extracts (ME) of P. lentiscus on fructosamine inhibition percent, AG: Amino Guanidine (1 mg/mL). Each value represents the mean of three replicates, and the bars represent the standard error. Different letters in each column indicate statistically significant differences at p < 0.05.

In this study, the inhibitory effect on fructosamine levels is shown in Figure 9. The extracts obtained from *P. lentiscus* demonstrated a significant dose-dependent reduction in fructosamine levels. At 1 mg/mL concentration, fructosamine levels were reduced by 17% and 27% for the aqueous and methanolic extract, respectively. Comparing these results to the positive control at a concentration of 1 mg/mL, our extracts showed lower values, with aminoguanidine exhibiting an inhibition rate of 89.89%. The ability of *P. lentiscus* extracts to inhibit fructosamine formation suggests they can intervene early in glycation, potentially slowing diabetes-related complications. Lowering fructosamine levels is linked to better postprandial glucose regulation and a reduced risk of vascular damage, highlighting its importance in diabetes management (Elrherabi et al., 2024).

**FIGURE 10:**
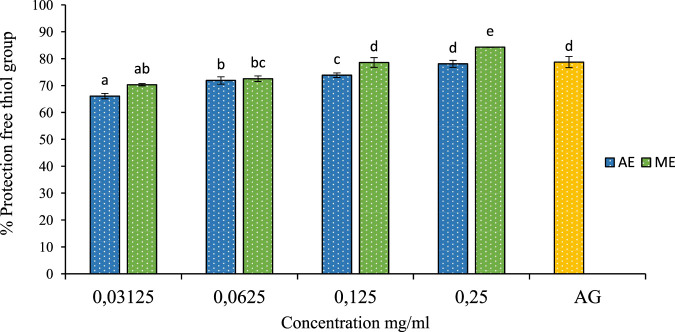
The effect of aqueous extracts (AE) and methanolic extracts (ME) of P. lentiscus on the percentage protection of thiol group, AG: Amino Guanidine (1 mg/mL). Each value represents the mean of three replicates, and the bars represent the standard error. Different letters in each column indicate statistically significant differences at p < 0.05.

The thiol group (R-SH) is a chemical functional group found in many organic compounds and biomolecules including proteins, enzymes, and antioxidants. Thiols are particularly important due to their reactivity and ability to form covalent bonds with other molecules, they play a crucial role in maintaining cellular homeostasis, acting as redox buffers that regulate oxidative stress, enzyme activity, and signal transduction pathways (González-Tortuero and Rodríguez-Rojas, 2023). However, Thiols can be sensitive to factors such as oxidation, dehydration, and reactive agents. Preservation thiol group involves protecting this function from undesirable reactions or degradations (Loi et al., 2015). In this study, we investigated the protective effect of *P. lentiscus* leaf extracts on free thiol groups. The results, shown in Figure 10, reveal that the extracts significantly protect thiol groups. The optimal concentration for this effect was 0.25 mg/mL of the aqueous extract, preserving 84% of free thiol groups. In comparison, 1 mg/mL of aminoguanidine provided 78% protection. This demonstrates that the aqueous extract of *P. lentiscus* is more effective at preserving thiol groups than aminoguanidine.

## 4 Discussion

### 4.1 Total phenolic and flavonoid content

The efficiency of phenolic and flavonoid compound extraction varies with solvent type due to differences in polarity, solubility, and interaction with plant matrices. Water, being highly polar, effectively extracts hydrophilic phenolic acids, flavonoid glycosides, and tannins, though it may also co-extract unwanted polar compounds like sugars and proteins. Methanol, with intermediate polarity, is more efficient at extracting a broader range of phenolics, making it a preferred solvent in phytochemical studies. The ability of these solvents to dissolve phenolics is influenced by their capacity for hydrogen bonding with hydroxyl (-OH) groups, with water forming the strongest hydrogen bonds, followed by methanol and ethanol. Additionally, extraction efficiency depends on temperature and methodology, as higher temperatures can enhance solubility but may degrade heat-sensitive phenolics. Therefore, solvent selection is crucial for optimizing the extraction of bioactive compounds, with methanol generally providing the best balance between yield and diversity of extracted phenolics (Dahmoune et al., 2014).

The variations in phenolic content among different studies may be attributed to multiple factors, including geographical origin, environmental conditions, plant maturity, and post-harvest treatment (El Ahmadi et al., 2024a). For instance, a study by Salhi et al. (2019) reported higher polyphenol levels in *P. lentiscus* collected from different Mediterranean regions, which could be linked to differences in climate, soil composition, and UV exposure, as these factors directly influence secondary metabolite production. Furthermore, drying and storage methods significantly impact phenolic stability, with higher degradation rates observed in prolonged exposure to light and heat. These findings emphasize the importance of standardizing extraction conditions and storage protocols to ensure consistency in bioactive compound yield and bioactivity (Bouabid, 2020; Calliste et al., 2001).

### 4.2 Qualitative identification of chemical composition of extracts

HPLC analysis identified four predominant phenolic compounds: gallic acid, quercetin, catechin, and myricetin, all of which have well-established antioxidant and metabolic regulatory properties. The predominance of quercetin and gallic acid aligns with findings from previous studies on *P. lentiscus* and related species, where these compounds have been recognized for their strong antioxidant, anti-inflammatory, and metabolic modulatory effects. Quercetin is particularly relevant in the context of diabetes management, as it has been reported to enhance glucose uptake in skeletal muscle, improve insulin sensitivity, and reduce pancreatic β-cell apoptosis (Dhanya, 2022). Additionally, catechin has also been reported to improve glucose metabolism and insulin sensitivity, highlighting its antidiabetic properties. Similarly, traces of syringic acid contribute to the plant’s therapeutic profile, as this compound is known to alleviate oxidative damage, regulate inflammatory pathways, and provide metabolic benefits. Collectively, these findings reinforce *P. lentiscus* as a valuable natural source of polyphenols with potential applications in managing oxidative stress and diabetes (Zhang and Tsao, 2016). These findings reinforce the pharmacological potential of *P. lentiscus*, providing a phytochemical rationale for its traditional medicinal use.

### 4.3 Antioxidant activity

The antioxidant capacity of *P*. *lentiscus* extracts was evaluated using four complementary assays: DPPH, ABTS, FRAP, and iron chelation. The aqueous extract exhibited superior radical-scavenging potential, with IC_50_ values significantly lower than those of the methanolic extract, outperforming synthetic antioxidants such as BHA in DPPH assays. The strong antioxidant activity of *P. lentiscus* can be attributed to its high polyphenol and flavonoid content, as confirmed by phytochemical analysis. Gallic acid, quercetin, and catechin, previously identified in our extracts, are well-documented for their radical-scavenging properties. Their hydroxyl (-OH) and phenyl (-C_6_H_6_) groups enable efficient electron donation, stabilizing free radicals and preventing oxidative damage (Mehenni et al., 2016).

In addition to scavenging free radicals, flavonoids like quercetin play a crucial role in inhibiting lipid peroxidation and stabilizing metal ions, further enhancing antioxidant defense. The chelating activity of the extracts is particularly noteworthy (The aqueous extract demonstrated strong iron-binding capacity (IC_50_ = 0.47 ± 0.001 mg/mL), closely approaching that of EDTA), as transition metals such as Fe^2+^ and Cu^2+^ catalyze oxidative stress reactions, leading to lipid peroxidation and protein oxidation (Salhi et al., 2019). Polyphenols act synergistically, meaning their combined presence in *P. lentiscus* likely amplifies overall antioxidant capacity. Beyond their antioxidant function, these bioactive compounds also modulate oxidative pathways and chelate metal ions, offering protection against oxidative stress-related diseases such as diabetes, neurodegenerative disorders, and cardiovascular conditions. These findings reinforce the therapeutic potential of *P. lentiscus* as a natural antioxidant source with promising applications in disease prevention and management.

### 4.4 Inhibition of α-amylase

The α-amylase inhibition observed in this study highlights the potential postprandial glucose-lowering effect of *P. lentiscus* extracts. The aqueous extract demonstrated a stronger IC_50_ (2.291 ± 0.002 μg/mL) compared to the methanolic extract (IC_50_ = 2.889 ± 0.002 μg/mL), though both were less potent than acarbose (IC_50_ = 0.9 ± 0.013 μg/mL), a standard pharmaceutical α-amylase inhibitor. The strong enzyme-inhibitory ability of *P. lentiscus* leaf extracts is mainly attributed to their rich content of natural bioactive compounds, particularly gallic acid, quercetin, and catechin. These compounds interfere with α-amylase function through multiple mechanisms; One way is by competing with starch for the enzyme’s active site (Competitive Inhibition); quercetin and catechin, have structures similar to starch and can attach to the enzyme’s active site, preventing starch from binding. This slows down the breakdown of carbohydrates, leading to a gradual release of glucose into the blood, which helps control blood sugar levels after meals (Bouakline et al., 2024).

Additionally, these plant compounds can form stable complexes with α-amylase, further hindering its activity (Enzyme Conformational Change). Flavonoids like catechin and quercetin have been found to bind firmly to α-amylase, altering its conformation and reducing its ability to hydrolyze starch (Mahboob et al., 2023). Molecular docking studies have demonstrated that catechin, for instance, exhibits a binding energy of −7.631 kcal/mol with α-amylase, indicating a strong interaction that disrupts the enzyme’s function (Bouakline et al., 2024). Polyphenols, like Acide gallic also act through a different method called allosteric inhibition, where they bind to areas of the enzyme that are not the active site. This attachment causes changes in the enzyme’s structure, making it harder for starch to bind and slowing down the enzyme’s ability to work (Sun et al., 2024). Another important way *P. lentiscus* extracts work is by removing metal ions, such as calcium, that α-amylase needs to function properly. Polyphenols, particularly flavonoids and tannins, can bind to these metal ions, making them unavailable for the enzyme. Without these essential ions, the enzyme becomes unstable and less effective at breaking down starch (Floris et al., 2024). Unlike synthetic inhibitors such as acarbose, which typically only block the enzyme’s active site and are associated with gastrointestinal side effects (Sun et al., 2024).

Finally, the different bioactive compounds in *P. lentiscus* extracts work together to enhance their effect on α-amylase. The combination of flavonoids, tannins, and other natural molecules creates a stronger overall inhibition compared to synthetic drugs (Pekacar and Deliorman Orhan, 2022). plant-based compound inhibitors offer a multi-targeted approach, interfering with enzyme activity through multiple mechanisms, they are considered highly effective natural inhibitors, making them promising for managing blood sugar levels and controlling carbohydrate digestion (Saheed et al., 2022).

### 4.5 Anti-glycation activity

The aqueous extract of *P. lentiscus* exhibited strong antiglycation activity, inhibiting AGE formation by 98.23%, comparable to aminoguanidine (98.88%). This effect was confirmed through fructosamine reduction and thiol group preservation, highlighting its multi-targeted inhibition of glycation pathways. The presence of high content of polyphenols and flavonoids, contributes to this activity through carbonyl trapping, ROS scavenging, and metal chelation, mechanisms that prevent irreversible AGE accumulation and oxidative stress induced protein damage (El Ahmadi et al., 2024b; Yeh et al., 2017).

Chelation of transition metals such as iron and copper further enhances its antiglycation potential, as these metals catalyze oxidative glycation reactions. Similar findings in Dendrobium officinale extracts confirm that polyphenols reduce CML and pentosidine formation by blocking glycation-induced oxidative stress. These properties suggest *P. lentiscus* may be beneficial not only for diabetes management but also for vascular aging, neuroprotection, and skin glycation prevention (Zhang et al., 2021).

Given its significant activity, future studies should focus on *in vivo* validation, pharmacokinetics, and formulation improvements to optimize bioavailability and clinical efficacy. As a natural alternative to synthetic AGE inhibitors, *P. lentiscus* offers promising potential for metabolic and age-related disease prevention.

### 4.6 Study limitations and future research directions

While this study offers valuable findings, several limitations should be acknowledged. The *in vitro* nature of the assays does not fully capture the complexity of *in vivo* metabolic processes, where enzyme kinetics, systemic metabolism, and tissue absorption can affect bioactivity. As previous studies have shown, *in vitro* findings often require further validation through animal models to better understand the pharmacokinetics and pharmacodynamics of bioactive compounds in a biological system. Future research should incorporate *in vivo* experimentation to determine the real-world efficacy and safety profile of *P. lentiscus* bioactive compounds (Chassagne et al., 2021). Additionally, clinical validation remains a crucial gap. While the extracts demonstrated significant biological activity *in vitro*, their effectiveness in human populations is yet to be established. Clinical trials are necessary to determine dosage, bioavailability, and potential side effects. A well-structured clinical investigation would provide the necessary translational link between laboratory findings and therapeutic applications. Another challenge in phytochemical research is variability in the chemical composition of plant extracts. Factors such as geographic origin, climate, harvesting season, and extraction methods influence the phytochemical profile. To ensure reproducibility and therapeutic consistency, future studies should focus on standardizing extraction protocols and employing advanced phytochemical profiling techniques such as LC-MS and NMR spectroscopy (Ma et al., 2024; Wang et al., 2021).

Furthermore, while our study suggests potential mechanistic insights, the precise molecular pathways through which *P. lentiscus* exerts its effects remain unclear. Future research should focus on molecular docking and computational modeling to predict interactions between bioactive compounds and metabolic enzymes. Omics-based approaches, including proteomics and metabolomics, could provide a more detailed mechanistic understanding of its antioxidant and antidiabetic properties (Vitale et al., 2022). Comparative studies with standard pharmaceutical drugs are also warranted. While natural products offer promising therapeutic potential, assessing their efficacy relative to established synthetic drugs is crucial for their integration into mainstream medicine. Future research should conduct side-by-side comparisons between *P. lentiscus* extracts and conventional antidiabetic or antioxidant medications to evaluate their potency, safety profile, and therapeutic value (Prabhakar and Banerjee, 2020; Mahmoud et al., 2024; Romano and Tatonetti, 2019).Additionally, formulation challenges and long-term stability must be addressed. Developing nanoencapsulation or liposomal formulations could enhance bioavailability and targeted delivery of bioactive compounds, improving their therapeutic potential. Recent advances in pharmaceutical formulation science suggest that novel delivery systems could significantly impact the stability and absorption of plant-derived compounds, making them more effective in clinical applications.

## 5 Conclusion

The present study has demonstrated that using different extraction solvents significantly influences the content of phenolic compounds and flavonoids. The aqueous extract of *P. lentiscus* exhibited the highest phenolic content, correlating with its superior antioxidant capacity. The results indicate that the antioxidant activity of *P. lentiscus* leaves is primarily attributed to their rich phenolic composition. The aqueous extracts exhibited the most significant antioxidant activity in DPPH, ABTS, FRAP, and ferrous ion chelation assays. Additionally, our study revealed that *P. lentiscus* extracts are potent inhibitors of the diabetic enzyme alpha-amylase and advanced glycation end-product formation. These findings strongly suggest that *P. lentiscus* leaves could serve as a valuable natural source of antioxidants and may hold significant potential as a therapeutic agent for managing oxidative stress and preventing diabetes-related complications. Future studies should focus on clinical validation and advanced formulation strategies to harness the full potential of *P. lentiscus* in pharmaceutical and nutraceutical applications.

## Data Availability

The original contributions presented in the study are included in the article/supplementary material, further inquiries can be directed to the corresponding authors.
